# Analysis of *POFUT1* Gene Mutation in a Chinese Family with Dowling-Degos Disease

**DOI:** 10.1371/journal.pone.0104496

**Published:** 2014-08-26

**Authors:** Mingfei Chen, Yi Li, Hong Liu, Xi'an Fu, Yiongxiang Yu, Gongqi Yu, Chuan Wang, Fangfang Bao, Herty Liany, Zhenzhen Wang, Zhongxiang Shi, Dizhan Zhang, Guizhi Zhou, Jianjun Liu, Furen Zhang

**Affiliations:** 1 Institute of Dermatology and Department of Dermatology at No.1 Hospital, Anhui Medical University, Hefei, Anhui, China; 2 Shandong Provincial Institute of Dermatology and Venereology, Jinan, Shandong, China; 3 Department of Human Genetics, Genome Institute of Singapore, Singapore, Republic of Singapore; 4 Shandong Provincial Hospital for Skin Diseases, Jinan, Shandong, China; 5 Shandong Provincial Key Lab for Dermatovenereology, Jinan, Shandong, China; 6 Shandong Provincial Medical Center for Dermatovenereology, Jinan, Shandong, China; MOE Key Laboratory of Environment and Health, School of Public Health, Tongji Medical College, Huazhong University of Science and Technology, China

## Abstract

Dowling-Degos disease (DDD) is an autosomal dominant genodermatosis characterized by reticular pigmented anomaly mainly affecting flexures. Though *KRT5* has been identified to be the causal gene of DDD, the heterogeneity of this disease was displayed: for example, *POFUT1* and *POGLUT1* were recently identified and confirmed to be additional pathogenic genes of DDD. To identify other DDD causative genes, we performed genome-wide linkage and exome sequencing analyses in a multiplex Chinese DDD family, in which the *KRT5* mutation was absent. Only a novel 1-bp deletion (c.246+5delG) in *POFUT1* was found. No other novel mutation or this deletion was detected in *POFUT1* in a second DDD family and a sporadic DDD case by Sanger Sequencing. The result shows the genetic-heterogeneity and complexity of DDD and will contribute to the further understanding of DDD genotype/phenotype correlations and to the pathogenesis of this disease.

## Introduction

Dowling-Degos disease (DDD [MIM 179850]) is an autosomal dominant genodermatosis characterized by reticular pigmented anomaly mainly affecting flexures, such as the neck, axilla and areas below the breasts and groin [1]. In 2006,Betz et al. performed a genomewide linkage analysis of two German families and identified loss-of-function mutations in the keratin 5 gene (*KRT5*) [2]. However, no *KRT5* mutations were identified in more than 50% of DDD familial cases and sporadic cases [3], [15], suggesting the genetic heterogeneity of DDD.

Recently, next generation sequencing technologies, including whole genome and whole exome sequencing, have been successfully applied to human genetics research to identify pathogenic genes []–[6]. Equipped with this advanced technology, two genes, *POFUT1* [7] and *POGLUT1* [15], were identified and confirmed to be additional pathogenic genes of DDD during the past year.

Here, we investigated two DDD families and one sporadic DDD case which are absent from *KRT5* mutations and performed genome-wide linkage and exome sequencing analyses in one DDD family. Only a novel mutation, c.246+5delG, in *POFUT* was identified to be potentially causal. This mutation was confirmed by Sanger sequencing to be present in all affected family members, absent in unaffected individuals that were sequenced except that the unaffected III8 in the family also carries it. III8, whose mother is a DDD case, is 12 years old and probably under the disease onset age. Sanger sequencing did not detect other novel mutations nor this deletion in *POFUT1* in a second DDD family and a sporadic DDD case. The result shows the genetic heterogeneity and complexity of DDD.

## Materials and Methods

### Subjects

**Figure 1:**
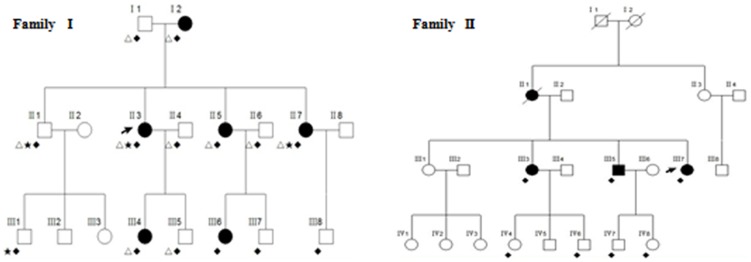
Family trees of Family I and Family II. Shown are the pedigree of family I and family II with DDD. The arrow denotes the proband; “Δ” denotes the individuals used in the linkage analysis; “★” denotes the individuals used in exome sequencing analysis, “⧫” denotes the individuals that were Sanger sequenced for *POFUT1*.

**Figure 2:**
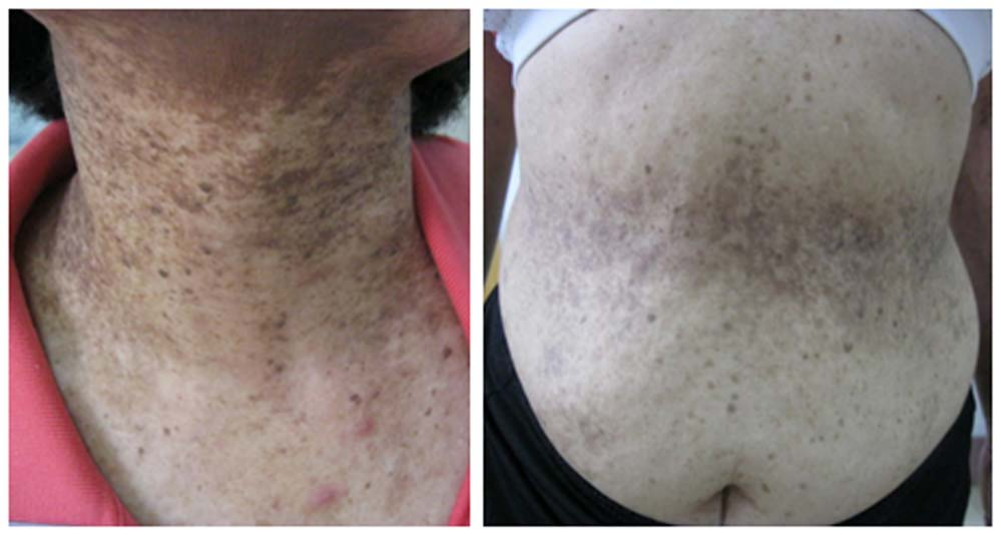
Clinical manifestations of DDD. Reticular hyperpigmentation on the skin of neck and abdomen.

Our study recruited two unrelated DDD families and one sporadic DDD case of Chinese ethnicity, totaling 10 affected and 12 unaffected individuals (Figure 1). In addition, 100 unrelated healthy controls of Chinese ethnicity were also Sanger sequenced. All patients were carefully examined by at least two experienced dermatologists, and were diagnosed by clinical features (Figure 2) and histopathological findings (Figure S1). EDTA anticoagulated venous blood samples were collected from all participants. Genomic DNA was extracted from peripheral blood lymphocytes by standard procedures using FlexiGene DNA kits (Qiagen).

The study was approved by the institutional review board at Shandong Provincial Institute of Dermatology. Written informed consent was obtained from all participants, or their guardian.

### Genome-wide linkage and exome sequencing analyses

Approximately 200 ng of genomic DNA was used for genotyping by Illumina Human 660W-Quad BeadChip for each of 10 individuals (Figure 1) in Family I. Multipoint parametric linkage analysis were performed in Merlin [8] by using the LD pruned autosomal SNPs (with LD<0.1 in population data) and assuming a dominant inheritance mode with a disease allele frequency of 0.001.

To pinpoint the causal mutations for DDD, exome capture was carried out using Agilent SureSelect Human All Exon Kit according to the manufacturer's protocols in two affected (II 3 and II 7) and two unaffected individuals (II 1 and III 1) in family I (Figure 1).

Each captured library was loaded on a HiSeq 2000 platform, and paired-end sequencing was performed with read lengths of 100 bp. Variants were called and filtered based on the “best practice variant detection with GATK (v3), that is “QD<2.0”, “MQ<40.0”, “FS>60.0”, “HaplotypeScore >13.0”, “MQRankSum <−12.5”, “ReadPosRankSum <−8.0” for single nucleotide variants (SNV), and “QD<2.0”, “ReadPosRankSum <−20.0”, “InbreedingCoeff <−0.8”, “FS>200.0” for indel.

### Sanger sequencing

All seven coding exons including intron–exon boundaries as well as 5′ UTRs and 3′ UTRs of *POFUT1* were amplified by polymerase chain reaction (PCR) using the published primers [7]. After amplification, products were purified and sequenced on ABI 3130xl Genetic Analyser. Mutations were identified by comparing with the reported DNA reference sequence (GenBank accession number: NG_032110). All the identified mutations were verified by the subsequent opposite-direction sequencing. Both the patients and control samples were analyzed using the same protocol.

## Results

### Linkage analysis

We firstly performed genome-wide linkage analyses in family I to determine the locus that co-segregates with the disease. Maximal LOD score of 1.5 was obtained on chromosome 8 and 20 (Figure S2). Haplotype co-segregation analyses by Haplopainter [9] mapped the linkage critical region to chr20: 17,722,478–36,009,145. There are 165 SNPs in chromosome 20 for linkage analysis after LD pruning.

### Exome sequencing analysis

Sequence reads were mapped by BWA [18] to hg19 human reference genome. On average, each sample had a total of *102063181* uniquely mapped reads (Table S1). Each sample had a mean coverage of 115.85 and 91% of the 50 Mb target regions were covered at 10× or more (Figure S3 and Table S2). GATK Unified Genotyper with the recommended filtering criteria was invoked to call single nucleotide variants (SNV) and indels. For SNV, 18,606 coding variants were identified on average in each individual (Table S3). The percentage of novel SNVs (not in dbSNP132) was 6.2%; Ti/Tv ratios for novel SNVs and SNVs in dbSNP132 were 2.90 and 3.22 respectively. 2215 SNVs were shared by the two patients and absent in the two healthy controls; 81 of which were not in 1000 Genome Project (Phase I, Oct 2012), none of them lied in the linkage critical region (Table S4).

5421 indels were identified on average in each individual. 170 were shared by the two patients and absent in the two healthy controls, 98 of which were not in 1000 Genome Phase I data (Oct 2012). Six out of these 98 indels lied in the linkage critical region, one of which is functional. This indel (c.246+5delG), 5 bp downstream of exon 2 in *POFUT1*, is a non-canonical donor splice site (within 3–8 bp of the exon-intron boundary).

### Sanger sequencing

To verify the identified mutation, we screened *POFUT1* coding sequences including intron-exon boundaries as well as 5′ UTRs and 3′ UTRs by Sanger sequencing most of DDD Family I, part of Family II members and one sporadic case (10 affected and 12 unaffected individuals together, Figure 1).

**Figure 3:**
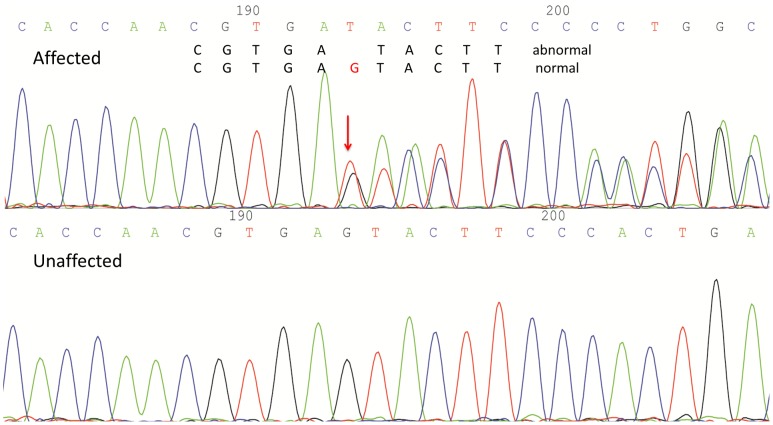
The 1-bp deletion in *POFUT1*. The Sanger sequencing traces around the position of 1-bp deletion c.246+5delG in one affected and one unaffected individuals of Family I.

In Family I, the novel deletion (c.246+5delG) in intron 2 of *POFUT1* was present in all affected and absent in all unaffected members that were sequenced (Figure 3), except that unaffected III8 carried it (Figure 1). However, III8, whose mother is a DDD case, is 12 years old and may well be under disease onset age (the average age at onset in Family I is about 20). This indel was absent in 100 unrelated healthy individuals of Chinese ethnicity that were Sanger sequenced. No other novel mutations or this mutation of *POFUT1* were identified in Family II and the sporadic case.

## Discussion

DDD is a relatively uncommon papillomatous pigmentary defect showing autosomal-dominant inheritance. It was first described by Dowling and Freudenthal in 1938 [10] and was termed by Degos and Ossipowski in 1954 [11].

After Betz et al. detected *KRT5* as a DDD pathogenic gene in 2006 [2], Li et al identified *POFUT1* as a new causal gene in 2013 [7]. This verified the genetic heterogeneity of DDD. *POFUT1*, located at 20q11, encodes two isoforms due to the alternative splicing. Researches have confirmed that this gene is an essential component of the canonical Notch signaling pathway [7], [12], which plays an important role in the regulation of melanocyte lineage development [13], [14].

Our linkage critical region (chr20: 17,722,478–36,009,145) includes gene *POFUT1*. Two variants were identified in *POFUT1* in our exome sequencing data of Family I – one was a SNV (chr20:30795819) which did not co-segregate with the disease, the other was an indel (c.246+5delG) in intron 2 of *POFUT1*. This indel is different from the two mutations identified by Li *et al* [7] which lie in exon 4 of *POFUT1.* c.246+5delG, 5 bp downstream of exon 2, is a non-canonical donor splice site (within 3–8 bases of the exon-intron boundary). We invoked NetGene2 [16] and MaxEntScan [17] splice site prediction softwares to investigate the effect of the 1-bp deletion on the splicing. The scores for the reference sequence around exon 2-intron 2 boundary from NetGene2 and MaxExtScan were 0.86 (> threshold of 0.5) and 9.54 respectively, while the scores for the 1-bp deletion sequence were below the threshold and −2.88 respectively, suggesting that the deletion might prevent the splicing between exon 2 and intron 2. Conservation scores and multiple alignments around the deletion from UCSC genome browser (Fig. S4) showed that the position affected by the deletion was conserved across species.

Recently, Basmanav et al [15] performed exome sequencing in five unrelated DDD individuals who were absent from mutations in *KRT5* and identified *POGLUT1* as another DDD causal gene. Only 2 SNPs in *POGLUT1* were identified in our exome sequencing data of Family I – one was SNV chr3:119196334 which was present in one unaffected and two affected individuals, absent in the other unaffected individuals. The other was SNV chr3:119198873 which was present in one unaffected and one affected individuals, absent in the other unaffected and affected individuals. Furthermore, *POGLUT1* is not in our linkage critical region, rendering the chance of *POGLUT1* being causal for DDD in Family I slim.

We should have genotyped more samples in Family I to increase the statistical power of the linkage analysis. However, it may not change the identified causal gene and mutation as the linkage analysis of Family I in our study and that of the discovery family in Li et al [7] pointed to the similar linkage critical regions.

In conclusion, our result further verified the genetic heterogeneity and complexity of DDD and contributes to the additional understanding of DDD genotype/phenotype correlations.

## Supporting Information

Figure S1 **Histopathological findings for DDD.** The lesion showed hyperkeratosis and filiform epithelial downgrowth of epidermal rete ridges. There were a large amount of melanins in the basal layer and some melanophages and melanins in the upper dermis. Left: HE 40×, right: HE 400×.
(TIF)

Figure S2 **LOD score of parametric linkage analysis vs genetic map for 22 chromosomes.**
(TIF)

Figure S3 **Percentage of the target regions covered at 5×, 10×, 20×, etc for the four individuals used in exome sequencing analysis.**
(TIF)

Figure S4 **Conservation scores and multiple alignments from chr20: 30,797,995–30,798,005 (around deletion c.246+5delG) by UCSC genome browser.**
(TIF)

Table S1 **Reads information for four exome sequenced individuals**.
(PDF)

Table S2 **Percentage of the target regions covered at 5×, 10×, 20×, etc for the four individuals used in exome sequencing analysis.**
(PDF)

Table S3 **Variant information for four exome sequenced individuals.**
(PDF)

Table S4 **81 SNVs identified in exome sequencing analysis that were shared in two DDD cases, absent in two unaffected controls, and not present in 1000 Genome Project (Oct 2012).**
(PDF)
