# Supplementary figures and images for: Analysis of POFUT1 Gene Mutation in a Chinese Family with Dowling-Degos Disease
Source: PLoS One. 2014 Aug 26;9(8):e104496. doi: 10.1371/journal.pone.0104496 (PMC4144801; doi:10.1371/journal.pone.0104496)

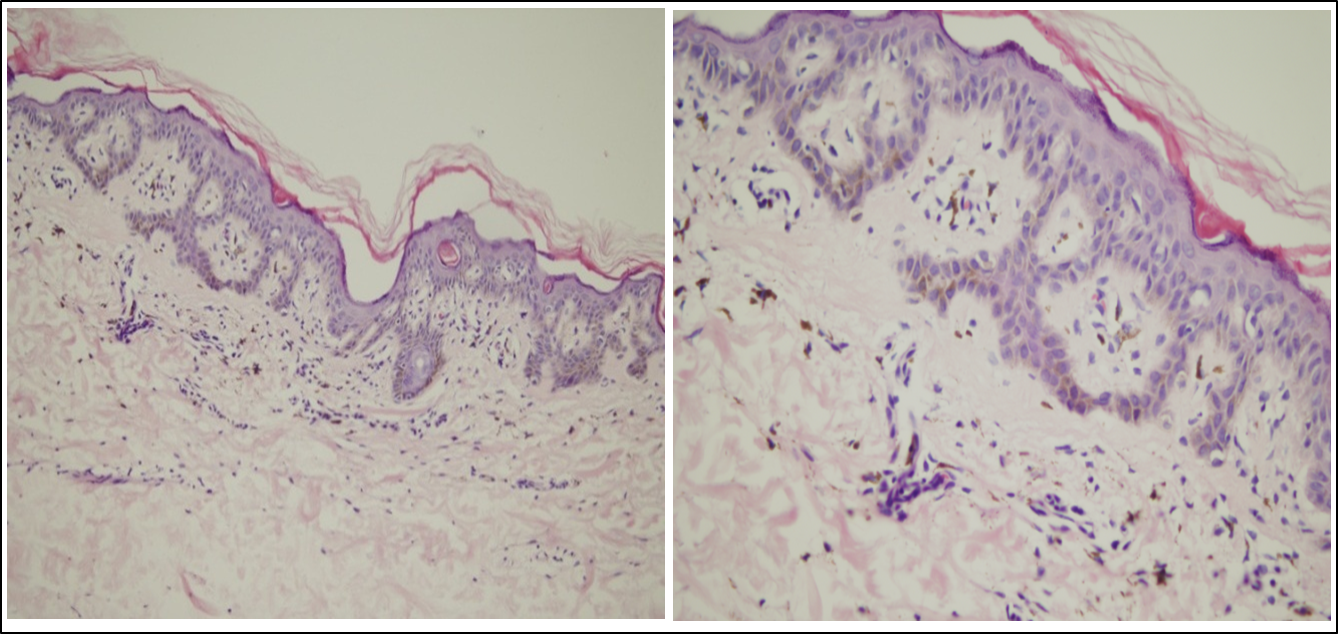

Supplement: Figure S1 — Histopathological findings for DDD. The lesion showed hyperkeratosis and filiform epithelial downgrowth of epidermal rete ridges. There were a large amount of melanins in the basal layer and some melanophages and melanins in the upper dermis. Left: HE 40×, right: HE 400×. (TIF) [file pone.0104496.s001.tif]

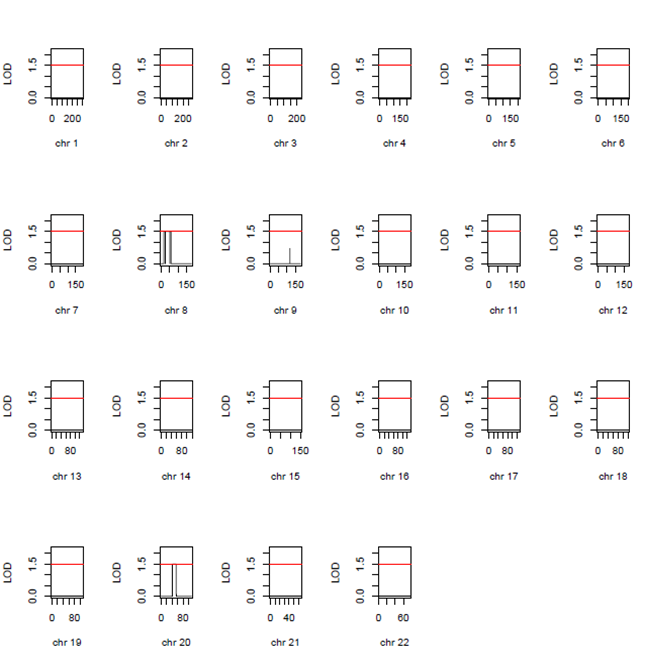

Supplement: Figure S2 — LOD score of parametric linkage analysis vs genetic map for 22 chromosomes. (TIF) [file pone.0104496.s002.tif]

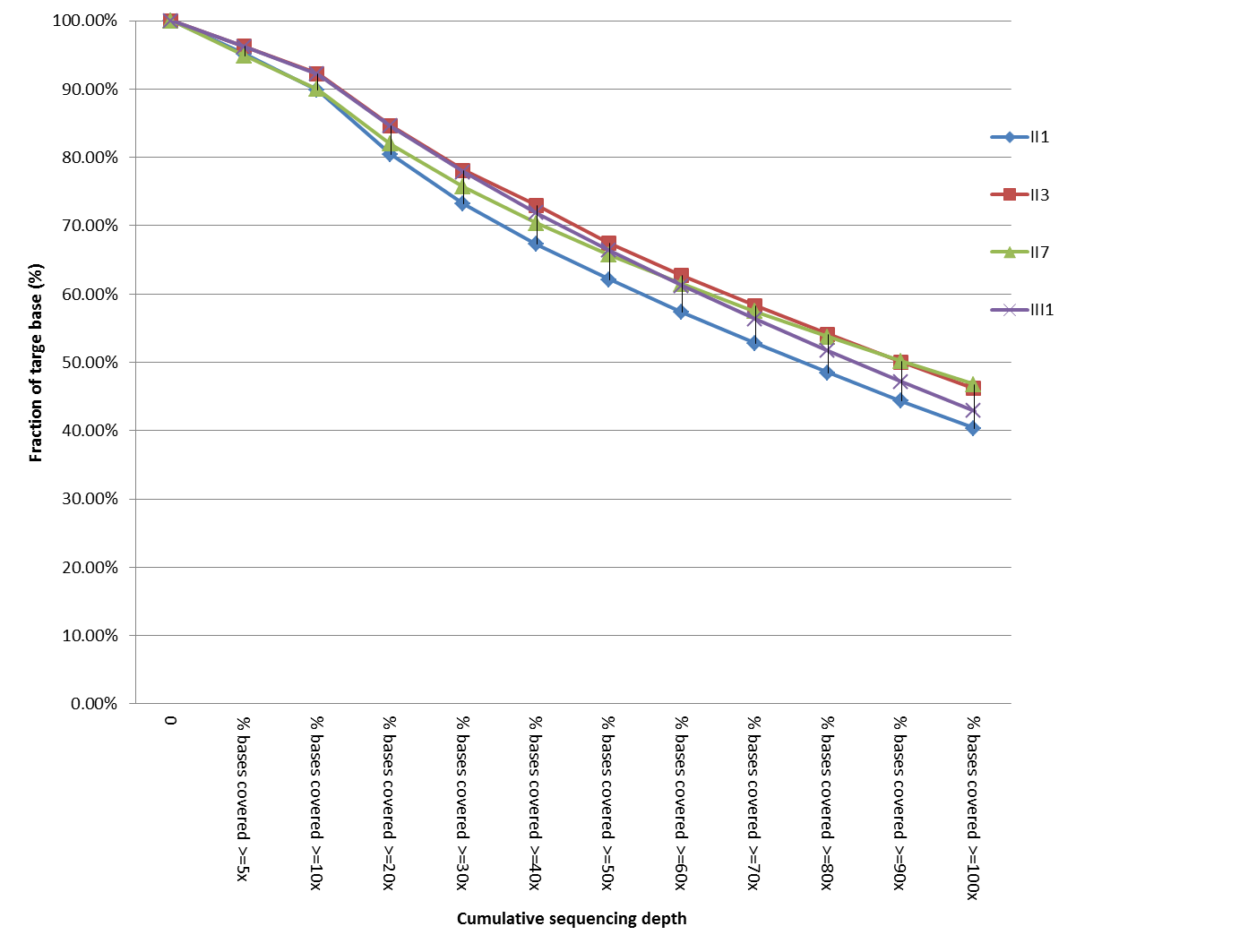

Supplement: Figure S3 — Percentage of the target regions covered at 5×, 10×, 20×, etc for the four individuals used in exome sequencing analysis. (TIF) [file pone.0104496.s003.tif]

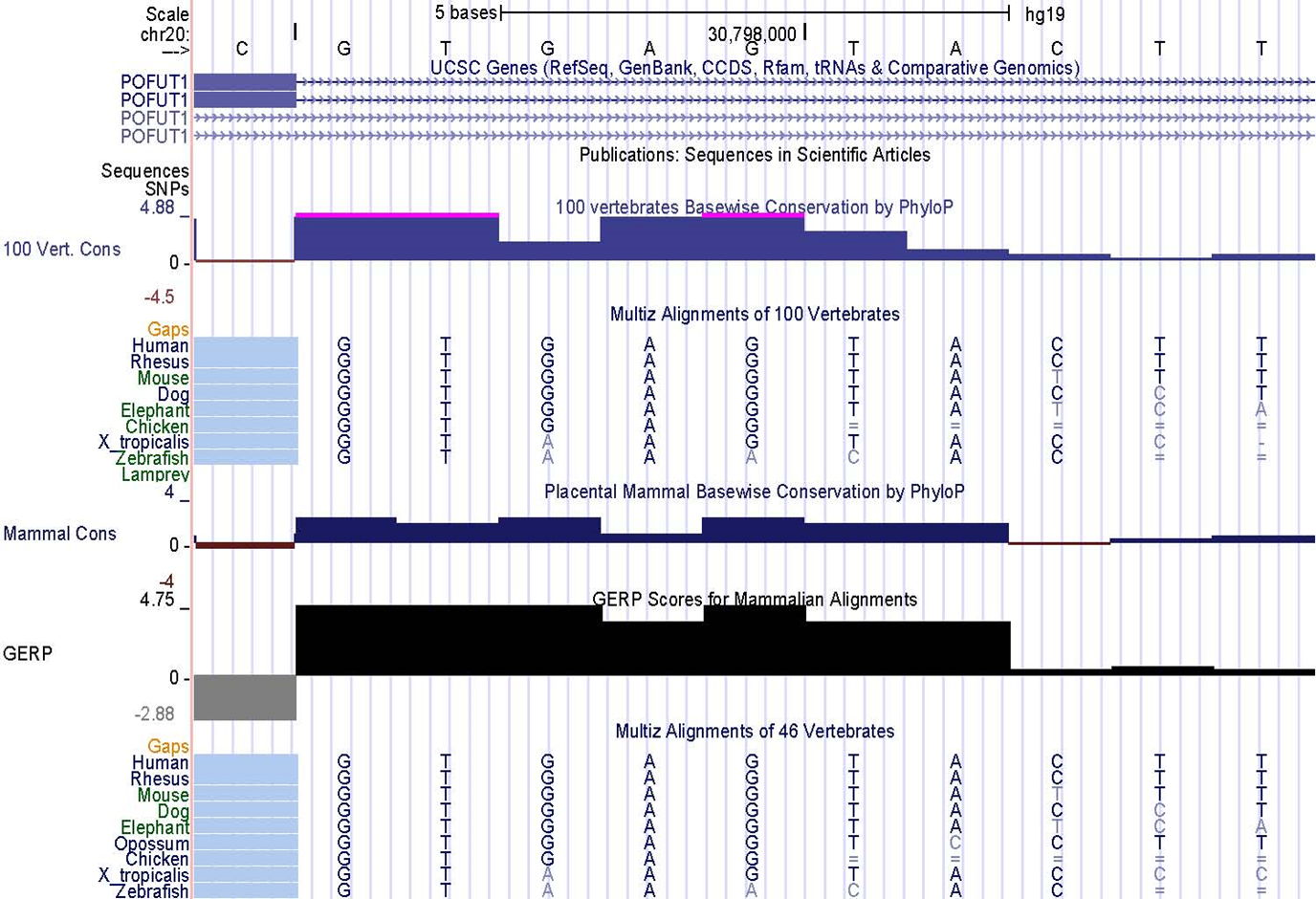

Supplement: Figure S4 — Conservation scores and multiple alignments from chr20: 30,797,995–30,798,005 (around deletion c.246+5delG) by UCSC genome browser. (TIF) [file pone.0104496.s004.tif]
