# Supplementary material for: Analysis of POFUT1 Gene Mutation in a Chinese Family with Dowling-Degos Disease
Source: PLoS One. 2014 Aug 26;9(8):e104496. doi: 10.1371/journal.pone.0104496 (PMC4144801; doi:10.1371/journal.pone.0104496)
Supplement: Table S1 — Reads information for four exome sequenced individuals. (PDF) [file pone.0104496.s005.pdf]

	**Ⅱ1**	**II3**	**II7**	**Ⅲ1**
Uniquely mapped, paired reads	91524940	111270190	108459553	96998042
Uniquely mapped, RF, FR reads	91412548	111151068	108277429	96892248
After removing PCR replicates	78524496	91335586	94578287	83339902
PCR redundancy	14.1%	17.83%	12.65%	13.99%
Mapped bases	5392872999	6261476324	6749884812	5734579987
Size in ROI	51797509	51797509	51797509	51797509
% bases in ROI	68.7%	68.6%	71.4%	68.8%
Mean	105.6	120.88	130.31	111.55
StDev	115.7	129.73	150.01	114.02
