# Supplementary material for: Analysis of POFUT1 Gene Mutation in a Chinese Family with Dowling-Degos Disease
Source: PLoS One. 2014 Aug 26;9(8):e104496. doi: 10.1371/journal.pone.0104496 (PMC4144801; doi:10.1371/journal.pone.0104496)
Supplement: Table S2 — Percentage of the target regions covered at 5×, 10×, 20×, etc for the four individuals used in exome sequencing analysis. (PDF) [file pone.0104496.s006.pdf]

depth	**II1**	**II3**	**II7**	**III1**
0	100.00%	100.00%	100.00%	100.00%
% bases covered >=5x	95.14%	96.24%	94.85%	96.21%
% bases covered >=10x	89.80%	92.24%	89.93%	92.17%
% bases covered >=20x	80.46%	84.61%	81.96%	84.57%
% bases covered >=30x	73.19%	78.09%	75.66%	77.85%
% bases covered >=40x	67.25%	72.97%	70.39%	71.85%
% bases covered >=50x	62.08%	67.38%	65.71%	66.34%
% bases covered >=60x	57.32%	62.72%	61.45%	61.21%
% bases covered >=70x	52.82%	58.33%	57.48%	56.32%
% bases covered >=80x	48.49%	54.12%	53.73%	51.65%
% bases covered >=90x	44.33%	50.06%	50.16%	47.17%
% bases covered >=100x	40.36%	46.16%	46.75%	42.92%
