# Supplementary material for: Analysis of POFUT1 Gene Mutation in a Chinese Family with Dowling-Degos Disease
Source: PLoS One. 2014 Aug 26;9(8):e104496. doi: 10.1371/journal.pone.0104496 (PMC4144801; doi:10.1371/journal.pone.0104496)
Supplement: Table S3 — Variant information for four exome sequenced individuals. (PDF) [file pone.0104496.s007.pdf]

	**II1**	**II3**	**II7**	**Ⅲ1**
Total number of SNPs	36051	36147	36151	35975
Synonymous	10199	10245	10259	10170
Missense	8914	9072	8982	8931
Nonsense	84	78	83	79
Splice site	286	279	293	304
Intron	12745	12651	12693	12668
5' UTRs	838	846	861	840
3' UTRs	1038	1040	1038	1043
Intergenic	1947	1936	1942	1940-
