# Supplementary material for: Analysis of POFUT1 Gene Mutation in a Chinese Family with Dowling-Degos Disease
Source: PLoS One. 2014 Aug 26;9(8):e104496. doi: 10.1371/journal.pone.0104496 (PMC4144801; doi:10.1371/journal.pone.0104496)
Supplement: Table S4 — 81 SNVs identified in exome sequencing analysis that were shared in two DDD cases, absent in two unaffected controls, and not present in 1000 Genome Project (Oct 2012). (PDF) [file pone.0104496.s008.pdf]

chr	position	ref	alt	Gene	chr	position	ref	alt	Gene
1	52704598	A	C	ZFYVE9	3	45963216	G	C	FYCO1
1	67263693	G	C	INSL5	3	48624944	G	C	COL7A1
11	120082064	C	A	OAF	3	110837786	A	G	PVRL3
11	130773154	G	C	SNX19	4	95162149	C	A	SMARCAD1
12	11244483	A	G	TAS2R43	4	113460680	C	A	AC023886.1
12	50571362	G	A	LIMA1	4	113460682	A	G	AC023886.1
12	57139851	A	G	PRIM1	4	126328128	C	T	FAT4
12	57573248	C	T	LRP1	4	152571733	C	A	FAM160A1
12	57624626	G	A	SHMT2	5	140795115	A	T	PCDHGC5
12	66698632	A	T	HELB	5	149576749	T	A	SLC6A7
12	69135644	C	T	NUP107	6	47254207	G	T	TNFRSF21
12	100720455	G	T	SCYL2	6	136552478	T	C	FAM54A
13	114325862	G	A	GRK1	6	146262798	G	A	SHPRH
14	90734740	C	T	PSMC1	7	43916458	G	A	URG4
14	96800828	T	C	ATG2B	7	51096965	C	T	COBL
14	99641781	G	A	BCL11B	7	65557823	T	C	ASL
14	100795099	C	T	C14orf68	7	72419420	A	C	POM121
15	40913557	G	T	CASC5	8	1497351	C	T	DLGAP2
15	78880748	C	A	CHRNA5	8	6566300	G	A	AGPAT5
16	11857414	T	C	ZC3H7A	8	11406628	A	G	BLK
16	58073895	A	T	MMP15	8	23560375	C	T	NKX2-6
16	61858972	G	A	CDH8	8	24207413	C	T	ADAM28
17	45009498	G	A	GOSR2	8	125571068	C	T	MTSS1
17	74075985	G	A	ZACN	9	71869325	A	G	TJP2
18	56586433	A	G	ZNF532	9	90501933	T	C	C9orf79
18	67231756	G	C	DOK6	9	92020309	C	T	SEMA4D
18	77896565	T	A	ADNP2	9	112030682	T	G	EPB41L4B

19	2334642	C	T	AC004410.1	9	116053844	C	T	PRPF4
19	3611261	G	A	C19orf29	9	116080895	C	T	WDR31
19	21607599	G	A	ZNF493	X	17746842	C	T	NHS
19	21719801	A	G	ZNF429	X	48136977	C	T	RP11-344N17.3
19	54759219	T	C	LILRB5	X	53114217	C	T	TSPYL2
19	54974053	C	T	LENG9	X	68749743	G	A	FAM155B
19	55998218	C	T	NAT14	X	77373561	G	C	PGK1
2	37035894	G	A	VIT	X	99931059	C	T	SYTL4
2	39212915	C	T	SOS1	X	101909416	T	C	GPRASP1
2	44571660	T	A	PREPL	X	140985188	C	T	MAGEC3
20	37150172	C	T	KIAA1219	Y	13309602	G	T	AC134878.1
20	37620141	G	A	DHX35	Y	13505584	A	C	AC134882.2
20	43052851	C	G	HNF4A	Y	13505618	T	C	AC134882.2
22	17073131	C	T	CESK1					
